# Highlights from Science Policy Interface sessions at the One Health Congress 2020

**DOI:** 10.1186/s42522-020-00033-4

**Published:** 2021-01-12

**Authors:** Chris Vanlangendonck, John Mackenzie, Ab Osterhaus

**Affiliations:** 1 One Health Platform, Berlare, Belgium; 2 Curtin University, Bentley, Australia; 3 Research Center of Emerging Infections and Zoonoses, Hannover, Germany

## Introduction

The 6th One Health Congress took place online between 30th October and 3rd November 2020 – the latter date coinciding with World One Health Day. The Congress was organised by the One Health Platform, which summarises here in this Meeting report its interpretation of the key messages from the individual talks at the meeting. The summaries are written entirely by members of the One Health Platform as indicated on the author list.

## AMR: challenges for scientists and policy makers

*An integrative approach to understanding AMR in New Zealand - development of a shared causal understanding - presented by Patricia Priest, University of Otago, New Zealand.*

One Health is an important lens through which to view antimicrobial resistance (AMR) because of the many factors involved: human, animal and environmental. However, relatively little work has been done to take a systems view and consider the feedback loops involved in AMR. For example, antimicrobials in livestock can be excreted and spread on the land, leach into rivers and streams, and potentially turn up in the drinking water of animals.

Feedback loops within the larger system can make it difficult to predict system behaviour, with sometimes unexpected and unintended consequences. A process was therefore started to model these different flows around a One Health system. The research aims to bring together human, animal and environmental health dimensions of AMR in New Zealand, and to model stakeholder understandings of the structure of the AMR system, with a focus on feedback loops.

The approach taken was System Dynamics Modelling which looks at feedback loops as reinforcing and balancing loops that essentially drive the behaviour of the overall system. In-depth interviews were carried out with participants from academia, research, policy, community, advocacy, industry, and clinical disciplines. Respondents were asked for their understanding of what drives AMR, and cognitive maps were generated leading to a set of causal loop diagrams.

The figure shows the causal loop diagram that incorporates all the feedback loops identified from the cognitive maps, and also incorporates a review of the literature on AMR system models.

The feedback loops were categorized in different loops. These were the development of antimicrobials and the pharmaceutical economics; the use of antimicrobials and food animals; collaboration; water quality; vulnerability to infection; the pressure to prescribe; and fear of germs.

This is the first participatory Systems Dynamic Model with professional stakeholders that integrates the human, animal and environmental aspects of AMR. Future research will involve triangulating the stakeholder model with the literature, setting up stakeholder workshops to refine and agree on a causal model, and producing a quantitative model to test leverage points and better understand the system.

*Capacities of African country to tackle global threat of antimicrobial resistance from the One Health perspectives - presented by Mohamed Moctar Mouiche Mouliom, ICF, Infectious Disease Detection and Surveillance (IDDS), Yaoundé, Cameroon.*

Joint External Evaluation (JEE) tools are used to assess a country’s capacity to combat AMR from a One Health perspective. JEE reports of 50 African countries were obtained, and four indicators of the AMR action packages were studied. These are AMR detection; infections caused by AMR pathogens; healthcare associated infection (HAI) prevention and control programmes; and antimicrobial stewardship activities. A scale from 1 to 5 was used to determine the level of implementation of each of these indicators in African countries.

The African countries were then grouped based on the similarity of the scores of the four indicators. Three clusters were identified: cluster 1 where 26 countries have no capacity; cluster 2 contained 17 countries with limited capacity; and cluster 3 included 7 countries with developed capacity. The latter were Zambia, Nigeria, Ethiopia, Kenya, South Africa, Uganda and Zimbabwe.

It is clear that Africa still has much to do in the fight against AMR. Implementation of a multi-sectoral AMR National Action Plan is vital to strengthen country AMR capacities. Inter-sectoral collaboration and communication should be encouraged to permit a One Health approach to address AMR.

There is a need for new strategies to encourage and increase commitment among country leaders towards improving health capacities. More sensitization and communication campaigns should be organised to create awareness and health education programmes at all levels in the community. WHO benchmarks for IHR capacities should be used by different countries to guide appropriate and effective measures and make progress in implementing One Health and AMR National Action Plans and measures to fight AMR at all levels.

Moreover, high-level political commitment is required for efficient implementation of prevention and control strategies against AMR. Also important is that the African Union (through the Africa CDC framework for AMR) endorses the African common position on AMR control strategies and finances AMR interventions. Finally, funding advocacy remains vital to support African AMR control strategies and to develop a monitoring and evaluation team to monitor progress.

*Pro- MED AMR - presented by Lawrence Madoff, International Society for Infectious Diseases, United States of America.*

ProMED (Program for Monitoring Emerging Diseases) began in 1994 as an electronic outbreak reporting system to monitor emerging infectious disease globally. It is intended and has been used as an early warning system for emerging disease outbreaks. The emphasis is on rapid reporting with a standard turnaround of less than 24 h. It’s freely available by subscription, with currently 85,000 subscribers. ProMED has a number of regional programmes throughout the world.

The traditional reporting process via public health has many advantages such as being robust, sensitive, accurate, validated and quantitative. However, it can be slow, with incentives for non-reporting, while broken links may also lead to non-reporting. It may miss uncharacterized or novel diseases, and is expensive. Event-based “informal” surveillance systems such as ProMED have many strengths. They can get reports from a number of sources such as ministries of health, health care workers, laboratories, media, lay public, WHO and local health officials, and can turnaround reports very quickly. Other benefits include transparency, the ability to identify any event, and low cost. Disadvantages are potential inaccuracy, the non-quantitative nature of the reports, and geographic bias. Since its inception, ProMED has embraced One Health.

The International Society for Infectious Diseases and ProMED have developed a new global surveillance network focusing on antimicrobial resistance (AMR). It will collect new information using digital detection methods and non-traditional sources which could be vetted, analysed and commented upon by a global team of AMR specialists. Reports on trends, new cases, clusters of AMR will be disseminated in real time to an international audience.

ProMED-AMR was launched in June 2020 in response to the growing worldwide threat of AMR. The increase in empiric use of antibiotics, driven by COVID-19, is anticipated to increase emergence of MDR nosocomial bacterial pathogens. The accumulation of resistance genes in the environmental microbiome from human, livestock, fish and plant overuse is inevitably transferred to human pathogens. At the same time, the development of new antibacterial drugs with novel mechanisms of action has been slow and limited.

*One Health for All: U.S. Strategies to Collaborate and Combat Antibiotic-Resistance - presented by Jomana Musmar, US Department of Health and Human Services, United States of America.*

In the US, more than 2.8 million illnesses and over 35,000 deaths result from AMR infections. The US is responding to AMR with a comprehensive and coordinated suite of actions implemented by a diverse set of agencies using a One Health approach. This started in 2013 with the CDC report on antibiotic resistance threats. The following year a Federal Task Force for Combating Antibiotic Resistant Bacteria (CARB) was established, and the Presidential Advisory Council on Combating Antibiotic Resistant Bacteria (PACCARB) was formed in 2015.

The PACCARB advises and provides information and recommendations to the Secretary of the US Department of Health and Human Services. Recommendations include programmes and policies intended to reduce or combat antibiotic-resistant bacteria that may present a public health threat, and improve capabilities to prevent, mitigate or treat such resistance. PACCARB holds 2–3 public meetings per year where public comment is welcomed, and over the years has prepared and submitted a number of key reports and recommendations:

Another initiative is the Federal CARB Task Force which pulls together relevant agencies across the One Health spectrum. It helped to develop the National Strategy for CARB in 2015 which guides the work of the Task Force. It is organised around five goals:

- Slow the emergence of antibiotic-resistant bacteria and prevent the spread of resistant infections.
- Strengthen national One Health surveillance efforts to combat resistance.
- Advance development and use of rapid and innovative diagnostic tests for identification and characterisation of resistant bacteria.
- Accelerate basic and applied research and development for new antibiotics, other therapeutics and vaccines.
- Improve international collaboration and capacities for antibiotic-resistance prevention, surveillance control, and antibiotic R&D.

The PACCARB has issued a report targeting priorities for the National Action Plan on combatting antibiotic-resistant bacteria 2020–2025. Many of the recommendations are continuations of the original Action Plan but with an emphasis on three novel areas: considerations for the environment, US global leadership in combating AMR, and drug-resistant fungi.

The CARB National Action Plan 2020–2025 presents a set of strategic actions to change the trajectory of AMR. It maintains the original goals and adds new objectives and targets. It continues to prioritise a One Health approach; infection prevention and control; the appropriate use of antibiotics; and support for innovative products to combat resistant infections. It enhances the focus on collecting data and turning it into useful information, and includes an updated measurement and reporting strategy.

## Addressing zoonotic diseases at the animal-human-ecosystem interface: responding to threats

*The One Health EJP network of European public organisations aims at reinforcing cross-sector preparedness - presented by Hein Imberechts, Sciensano, the Belgian Institute for Health.*

As microbes, including pathogens and resistance genes, move across sectors, a cross-sector One Health approach is essential to get better prepared, to manage, and to recover from disease urgencies that occur at the environment-animal-human interface. Zoonoses, foodborne infections and antibiotic resistance are the areas most likely to profit from a One Health approach. However, many barriers remain, while multidisciplinary is essential, incorporating input from social sciences, economics, biodiversity and other fields.

EU legislation supports cooperation between the European Commission and Member States, and initiatives in the One Health context already exist such as the FAO/OIE/WHO Tripartite, collaboration on AMR, ad hoc networks of ECDC and others. However, there is a need for practical, supportive guidelines and assistance to encourage cross-sector collaboration and coordination among partners involved in outbreaks that affect animal health, public health, safety, and the environment.

The One Health EJP Consortium brings together public organisations and has set up research projects based on a common research agenda and integrative activities to enable aligned and harmonised methodologies and procedures. It closely follows recommendations set out by the ECDC. Currently it incorporates 38 organisations from 19 EU countries. It has a 5-year budget of 90 million euros, 50% of which is funded by the EU. It includes 24 joint research and 5 joint integrative projects, while education and training is provided for 17 PhD studies as well as for 50+ short-term missions, workshops, summer schools and continuing professional development modules.

The objectives of the One Health EJP include developing and consolidating the European network of public organisations with reference laboratory functions on infectious diseases; integrating public health, animal health and scientists in the field of food and feed safety; and improving prevention, detection and response in the fields of foodborne zoonoses, AMR and emerging infectious threats. It comprises a number of work packages:

Ongoing research projects include:

- Foodborne zoonoses: NGS based methods, biosecurity interventions, tools for detection outbreaks, improved surveillance, source attribution, trends in human salmonellosis.
- AMR: Phenotypic methods, dynamics of AMR, epidemiology of AMR, risk assessment of AMR, tools for early detection.
- Emerging threats: Laboratory detection methods, non-NGS based methods, threat characterization.

An important outcome of the Science to Policy Work Package is the development of the One Health Inventory which is publicly available on the EJP website. It supports the dissemination of the results of the various activities.

*Integrating climate and environment public dataset in surveillance for early warning – presented by Maria Grazia Dente, National Centre for Global Health, Istituto Superiore di Sanità, Italy.*

Surveillance of vector-borne diseases is one of the best examples of diseases benefiting from the establishment of integrated systems in accordance with the One Health concept. Integrated surveillance systems for arboviruses have been implemented in a number of countries. However, in the majority of countries, this information is rarely shared in a timely manner between sectors to prevent outbreaks. An early-warning capacity therefore needs to be reinforced.

The framework of the MediLab Secure network, financed by the EU, has two specific action areas.

- Networking: to reinforce the capacity of countries in the Mediterranean and Sahel regions to prevent vector-borne diseases.
- Capacity building: to enhance laboratory preparedness and response capacities to arboviruses and their vectors, and enhance integrated surveillance, risk assessment and early warning to prevent and control epidemics and epizootics.

A key approach is to identify which indicators are feasible to collect and utilize in an early warning system that includes all the sectors involved in surveillance. In fact the identification of early warning indicators in association with the rapid implementation of prevention and control measures could reduce the severity of arbovirus epidemics. Ad hoc indicators can also highlight the vulnerability of countries or specific zones to the introduction and spread of arbovirus infections, thus providing precious information to prevent the occurrence of outbreaks and epidemics. With this aim in mind, a set of surveillance indicators were identified, focusing on seven emerging and re-emerging arboviruses.

The most frequently collected indicator in the entomology sector is “vector presence”, regardless of the involved pathogen. In the human sector, population density and age distribution are the most frequently collected indicators. For the animal sector, data on animal population/density are collected in 100% of cases for domesticated animals but rarely for wild species. This shows that relevant indicators are collected in each sector which can contribute to an integrated surveillance and early warning system for the prevention and control of arbovirus infection.

It was concluded that a certain grade of surveillance data (indicators) is already in place in the MediLab Secure region. Their collection should be strengthened and the gaps on critical indicators addressed (i.e. vector infection rate and wildlife information). A low level of use of global public datasets on climate and the environment in the study area was highlighted.

*Improvement of One Health collaboration at the national level for early warning, risk-assessment and control of emerging zoonotic diseases – presented by Catharina Maassen, National Institute for Public Health and the Environment, The Netherlands.*

It appears that animals do not have a major role in the transmission of SARS-CoV2 back to humans. But what if cats, mink, pigs or other animals would transmit the virus back to people? With this scenario in mind, it would be useful to organise signalling, assessment, response and control for emerging zoonoses in a One Health way.

The Netherlands has developed an integrated human-veterinary risk analysis structure for zoonoses. Tasks and responsibilities are arranged at the operational level as well as at the policy level with respect to risk assessment, risk management, and risk communication. Key is that in all steps, relevant sectors are represented. By bringing the information from the different sectors together in an early phase, the time to response could be minimized. And doing the different steps together leads to broad support for the actions and measures to take.

The project “COHESIVE: One Health Structure in Europe” was started with the aim to develop sustainable One Health approaches for the signalling, assessment, response and control of zoonoses at the national and regional level within EU countries. It seeks to support countries by setting up and strengthening One Health collaboration in the area of risk analysis of zoonoses by developing guidelines that focus on implementation and operationalisation.

Six activities were identified: signalling (early warning), risk assessment, feasibility assessment, risk management, risk communication, and governance. Some of these need to be done in a joint fashion; others in a coordinated way. A roadmap to implementation was developed, including steps such as goal-setting, statistical analysis, a workshop on system mapping, and plan building.

Barriers are always likely to arise, such as obtaining the political will, generating the necessary financial resources, cultural differences, conflicts of interest, privacy issues, sharing information etc. However, by building on trust and respectful relationships, and knowing each others’ roles and responsibilities in peace time, such an approach will definitely help in a crisis. And some governmental support would also be helpful!

*Beavers, Brexit and birch trees: what do the next 10 years have in store for wildlife and how will it impact the UK’s epidemiological landscape? – presented by Flavie Vial, Animal and Plant Health Agency, United Kingdom.*

Diseases involving wildlife as hosts pose significant issues for the health and well-being of people, livestock, companion animals, and wild animals themselves, and impact a country’s ability to trade internationally with a disease-free status. Examples of where a One Health approach is central to the UK’s Animal and Plant Health Agency include surveillance of the *Echinococcus multiocularis* tapeworm in foxes, the population dynamics of feral wild boar with respect to foot and mouth disease and swine fever outbreaks, and *Klebsiella pneumoniae* in common seals.

Climate change is likely to influence the avian influenza transmission cycle and directly affect virus survival outside the host. There is a potential increased risk of vector-borne diseases as temperatures rise. In the last few years the first cases of canine *Babesiosis* were identified, as well as an exotic tick species on a horse.

Beavers are a symbol of rewilding; the proposed restoration of ecosystems through the introduction of species. Rewilding is seen by many as the way to stem the loss of biodiversity and the functions and services that biodiversity provide to humanity. However, little is known about the risks associated with rewilding, and the potential natural or introduced diseases and pathogens when animals are (re)introduced. Monitoring the likelihood of novel introduction routes in wildlife is important, such as *Echinococcus multiocularis*, especially since the discovery of free-living racoon dogs in England, which are potential host species. A further example is the creation of new birch forests in the north of England, and its potential role in wildlife disease spread, control and management.

The risk of exotic diseases and invasive species impacting the UK is likely to increase after Brexit due to the wider range of trading partners the UK will be dealing with. Global container shipping has been identified as a major pathway for introducing species to new environments. Examples include bark beetles on timber, aquatic organisms in ballast water, and rodents and insects in grain shipments. It’s also essential to increase surveillance of non-native species of mosquitoes.

The One Health concept recognises the impact of ecosystem changes on human health. Biological invasions need to be framed in the context of a trans-disciplinary, socio-ecological system in which wide implications including health and socio-economic impacts are considered.

What are the likely implications of COVID-19 on the animal health field? In the short term, reduced human activity has seen increased suburban activity of wildlife, potentially increasing the risk of infection spillover. A decline in hunting (a non-essential activity) could result in insufficient population control of wildlife reservoir hosts. COVID-19 is also impacting the capacity of veterinary clinics and laboratories. This is of concern since horizon scanning indicates that several exotic animal diseases such as Rift Valley Fever are currently present at the borders of Europe.

On the other hand, COVID-19 has raised awareness among decision-makers and the public of the risk of novel disease transmission from wildlife; most acutely, of the links between wildlife exploitation, trade, and zoonotic disease transfer. Global wildlife experts are calling for improvements to how pathogens are tested and tracked in wildlife to reduce the risk of future pandemics.

## Empowering global health security and policy in Africa

*Operationalizing One Health in Liberia: Bringing Sectors Together for Resilient Health Services Post-Ebola – presented by Sonpon Blamo Sieh, National Public Health Institute of Liberia, Liberia.*

In Liberia, 19 human and 12 animal health epidemic-prone diseases are under surveillance, some of which (e.g. rabies, Lassa Fever, Ebola) are across sectors and so demand a collaborative cross-sectoral approach. This was particularly essential to respond effectively to the 2014–16 Ebola outbreak, during which the West African countries endorsed a One Health approach. This led to the development of the National One Health Strategic Plan in 2018 with objectives and strategic pillars for the adoption of the One Health approach in Liberia. It includes a One Health governance structure:

A One Health Coordination Platform provides the terms of reference to the various working groups and guides One Health interventions in Liberia. A number of joint interventions and activities have been implemented. These include joint planning meetings to generate buy-in of the One Health Coordination Platform; observance of global events; support of joint investigations; active case finding; and monthly Technical Working Groups (TWGs) consisting of members from human, animal and environmental health sectors.

Many challenges have been faced, such as joint planning and implementation. TWG members have competing priorities, although streamlining assessment and mapping partner resources can help to relieve this bottleneck. Mutual accountability and proactive project development is critical: process tracking and coordination meetings can help to align sectors and create feedback loops. There is a risk of system failure, with unstable or limited funding, combined with the unavailability of sustained essential emergency supplies such as no ribavirin to treat Lassa Fever patients; no human rabies vaccine; lack of animal rabies vaccine to reduce associated deaths from animal bites; no diarrheal kit; and irregular reagent supplies.

Challenges exist in other areas. Staff attrition and limited workforce limit IHR capacity; coordination needs the establishment of a functioning One Health structure and governance at sub-national level; evaluation tools are missing at the environmental health sector level; and lack of surveillance allows uncontrolled population movement within endemic districts and along borders with neighbouring countries. Moreover, there is a general absence of legislation, while specific areas are left unregulated. Having said that, the Regional Disease Surveillance Systems Enhancement (REDISSE) is allocating budget in five areas:

- Surveillance and information systems.
- Strengthening of laboratory capacity.
- Emergency preparedness and response.
- HR management for effective disease surveillance and epidemic preparedness.
- Institutional capacity building, project management, coordination and advocacy.

*Zoonotic disease preparedness in Sub-Saharan African countries – presented by Linzy Elton, University College London, United Kingdom.*

The Pan-African Network for Rapid Research, Response and Preparedness for Infectious Diseases Epidemics consortium consists of nine sub-Saharan African (SSA) and four European countries. A study was conducted to identify gaps in zoonotic disease preparedness strategies across SSA and implement research studies and training to address them. The survey was conducted using WHO’s Joint External Evaluations (JEE). The answers were grouped into Prevent, Detect, Respond, and Other, each of which has categories such as AMR, Zoonoses etc. These in turn have sub-categories such as Surveillance, Workforce, and Response, with technical questions that indicate technical preparedness. Each indicator is scored from 1 (no capacity) to 5 (sustainable capacity).

44 SSA countries have completed the JEE to date. A zoonotic disease preparedness score was calculated for each country, and data were weighted by region. The overall SSA score was 2.35 with a range of 1 to 4, and the mean country scores were indicated on a map:

The majority of countries had either limited or developed capacity. Two countries – Namibia and South Africa – had demonstrated or sustainable capacity. For the sub-categories, the mean SSA scores were: Surveillance 2.45; Veterinary Workforce 2.76; Response 1.84. By region, southern Africa scored highest in all three sub-categories, with Central Africa generally scoring the lowest.

Looking further at the technical questions that were asked for each sub-category, it’s possible to identify the percentage of countries across the continent that confirmed they had indicators in place. In the Surveillance sub-category, 77% of countries had a surveillance system in place for up to five zoonotic diseases, but only 30% had a system in place for more than five diseases. In the Veterinary Workforce sub-category, 70% of countries reported having veterinary worker training in place. In Response Mechanisms, 34% of countries reported having an inter-agency zoonotic response team, but only 9% reported that they had the capacity to respond to 80% of events in a timely manner.

Particularly interesting was the priority list of zoonotic pathogens. Just under half of SSA countries had a multi-sector approved list of five priority zoonotic diseases. The three most commonly cited diseases were rabies, anthrax and brucellosis. With almost 90% of countries citing rabies, a continent-wide plan with strong governmental backing and funding could have a significant impact. Some of the so-called emerging diseases include Ebola, West Nile Virus, and MERS-CoV, which are likely to occur in fewer countries.

To sum up, of the zoonotic disease preparedness categories, Veterinary Workforce had the highest SSA score. By utilizing and adapting the strengths from this category – training – the scores of other countries and regions could be improved. The Response sub-category had the lowest SSA score. Here, an indicator to target would be to improve communication on zoonotic diseases between different sectors.

The region that scored the highest was Southern Africa. By looking at how their strengths can be adapted to other countries, the scores of other countries could be improved, although it’s important to note that zoonotic diseases can be quite geographically distinct, and the policies will need to be strongly adapted.

*South-to-South Mentoring as a Vehicle for Implementing Sustainable Health Security in Africa – presented by Stephanie Marie Norlock, International Federation of Biosafety Associations (IFBA), Canada.*

The International Federation of Biosafety Associations (IFBA) is an NGO focusing on promoting safe, secure and responsible work with biological materials. Its Global Mentorship Program (IGMP) is a keystone in building health security capacity and promotes positive biosafety and biosecurity culture through multidisciplinary and multi-collateral dialogue as well as local contextualization of best practices in these areas. The regions of the world that are the most interested in the IGMP are on the African continent:

The IGMP is an exceptional opportunity for interactive and dynamic information exchange and an effective tool to communicate with national, regional and international scientists and biosafety professionals.

Examples in Morocco and Algeria include the biosecurity workshop “Engaging Young Scientists from the Global South in Biosecurity Diplomacy”, which was organised by the UN Office for Disarmament Affairs, and the workshop “MENA Regional Dialogue on High Containment Laboratories”. Two mentees from the IGMP were selected as national (Algerian) qualified professionals in biosafety to ensure inspection and selection of laboratory diagnosis of COVID-19. Local peer mentorship is an efficient way to develop necessary skills at the regional level and consolidate communication between regional networks of experts. Collaboration between biosafety professionals and decision-makers ensures input to policy development that is risk-based, locally driven and sustainable over the long-term. It also allows the development of harmonised and specific biosafety and biosecurity legislation, and provides immediate and effective outbreak management and emergency response. Such collaboration contributes to the definition of the adapted strategies and policies to ensure the sustainable development of global health security.

In Kenya, IGMP mentors give career talks and mentorship to high-school students and teach biosafety courses as part of the Kenya Laboratory Biorisk Management Curriculum which was launched in 2019. Other areas include laboratory waste management, occupational health and safety, and risk assessment. A Biosafety and Biosecurity Workshop in Nairobi was facilitated by experts from the Netherlands. Communication is still ongoing, sharing biosafety experiences during the COVID-19 pandemic. Discussions have also been held around One Health, and it was agreed that lab security needs to be defined differently to farm/field security, and an approach to biorisk management should be tailored to the specific context.

In South Africa, an IFBA certified professional in biorisk management and biosecurity sits on several committees including the council for the non-proliferation of weapons of mass destruction, and the biosafety and biosecurity JEE implementation technical working group. Being involved in the IGMP creates collaborations where partners supplement each other’s knowledge. It also allows a person to become a “sounding board” for new ideas and concepts that could potentially work in different settings.

*One Health Zoonotic Disease Prioritization for Multisectoral Engagement in the Economic Community of West African States (ECOWAS) – presented by Casey Barton Behravesh, Centers for Disease Control and Prevention (CDC), United States of America.*

The One Health Zoonotic Prioritization Process is transparent, allowing equal input from human, animal, and environmental health sectors. It is flexible and scalable and allows for local adaption. It prioritizes even in the absence of reliable prevalence data, while timely outcomes maximize impact, and outcomes can focus limited financial and personnel resources.

Workshops have been conducted at the national, sub-national and regional levels throughout the world. In Africa, 17 workshops have been conducted in partnership with different governments. In the Economic Community of West African States (ECOWAS) community, 15 Member States participated and were invited to send representatives from the Ministries of Health, Agriculture, and Environmental Health to serve as voting members.

One goal of the ECOWAS workshop is to use a multisectoral One Health approach to prioritize endemic and emerging zoonotic diseases of greatest regional concern that should be jointly addressed by human, animal and environmental health ministries and other partners using a One Health approach. A second goal was to develop next steps and action plans for addressing prioritized zoonotic disease through a multisectoral One Health approach.

Criteria were selected for ranking zoonotic diseases. These covered severity of disease; prevention and control capacity; epidemic and pandemic potential; ability to detect the disease; and socio-economic and environmental impact. Specific questions per criteria were asked. It resulted in a list of seven priority zoonotic diseases for ECOWAS: anthrax, rabies, Ebola and other hemorrhagic fevers, zoonotic influenza, zoonotic tuberculosis, trypanosomiasis, and yellow fever.

In terms of next steps and recommendations, one focus area was on One Health coordination. ECOWAS participants wanted to establish or continue to support a sustainable, national One Health platform or coordinating mechanism in each country. They wanted to increase engagement in the environmental health sector in One Health activities in the region, and establish governance documentation and a One Health strategic plan. A further recommendation was for engagement of political leadership in high-profile assessment and planning initiatives.

Regarding Surveillance and Laboratory, ECOWAS Member States consider it essential to strengthen existing Regional Animal Health Centres and the West African Health Organisation network. They also want to strengthen existing laboratory networks and diagnostic capacity.

In the area of Response, Preparedness, Prevention and Control, recommendations are to develop response plans for all priority zoonotic diseases using a One Health approach; harmonize response plans between neighbouring Member States and establish agreements for cross-border assistance; consider rostering One Health rapid response teams; harmonize sub-regional and regional legislative efforts for preventive and control efforts; and synergize rabies control programs and vaccine requisitions.

*Programming for Global Health Security: Challenges and Pathways to Resolution – presented byJeff Mecaskey, DAI Global Health.*

Tackling Deadly Diseases in Africa (TDDA) is the UK’s flagship programme for health security in Africa. It coordinates closely with WHO/AFRO, focuses largely on Cameroon, Chad, Ivory Coast, Mali, Niger and Uganda, and scopes its support according to fundamental technical norms.

The aim of TDDA’s Problem-Driven Political Economy Analysis (PEA) is to characterize the basic problems behind performance gaps in health security and identify strategies for their resolution. It involves analysing stakeholder roles, activities and priorities; identifying major political, economic and other contextual forces; assessing how these forces affect stakeholders; and identifying strategies for strengthening institutional, systems and individual capacities necessary for improving functional performance. The PEA identified a range of underlying issues beyond technical capacity challenges:

- Poorly regulated governance and limited political incentives to invest in health security.
- Limited accountability and lack of social pressure on governments to invest in health security.
- Limited organisational capacity and institutional fragmentation.
- Poorly harmonised international funding systems, reinforcing the silo of health security isolated from routine planning and budgeting.
- Conflict and insecurity leading to limited state control over territory and borders.

Key problems were identified across all the countries, as well as the country-specific ideology or origin for those problems.

The PEA highlights the importance of systems, institutions and governance in health security/One Health and the priorities for their strengthening. It underscores the role of donors/official development assistance in institutional fragmentation, with health security/One Health siloed from routine policy planning and budgeting. It also sheds light on the importance of conflict and insecurity in Sahel countries, e.g. the recent coup d’état in Mali.

These discoveries lead to a set of implications. Firstly, health security and One Health are technically defined, but to generate wider political support it’s necessary to reframe discussions in practical language. Line ministries (e.g. animal, environmental and human health) each have their own purposes: effective cooperation depends on negotiating understanding about shared value and purpose. Moreover, health security/One Health remains largely donor financed; embedding in national planning and budgeting could increase capacity for functional performance including prospects for Domestic Resource Mobilisation (DRM). Finally, health security/One Health are about the Sustainability Development Goals and about health and equity: if the system cannot deliver a poor woman’s baby safely, how is it going to deliver on epidemic preparedness and response?

## Sustainable approaches to one health

*The Vital Importance of a Web of Prevention for Effective Biosafety and Biosecurity in the twenty-first Century- presented by Tatyana Novossiolova, Center for the Study of Democracy, Bulgaria.*

The multi-faceted global impact that the COVID-19 outbreak has had on virtually every sphere of social life demonstrates that biological threats – regardless of their origins – can have far-reaching consequences. COVID-19 has thus highlighted the need to strengthen national and international disease prevention, detection, preparedness and response systems. Strengthening these systems requires the adoption of a holistic approach to the prevention of biological threats. It is helpful to think of this required approach as an integrated and comprehensive “web of prevention” in which all the key elements complement and reinforce each other to create an effective counter to the threat of disease, regardless of its origins.

The proposed model framework groups the different strands of this web of prevention framework into two categories: international biosafety instruments and international biosecurity instruments. These are grouped based on their primary purpose: whether they aim to prevent the unintentional or accidental release of biological agents and toxins (biosafety), or their deliberate release (biosecurity).

International biosafety instruments cover biodiversity perseveration, safe handling of biological agents and toxins, and health and food security. International biosecurity instruments cover four interconnected areas: weapon prevention, countering illicit trafficking, detection and investigation, and physical security & responsible conduct.

Addressing the complex picture of biological risks in the twenty-first century requires the full and effective implementation of both international biosafety and biosecurity instruments. The web of prevention can serve as a valuable conceptual framework for developing an understanding of the mutually reinforcing relationship of these two sets of instruments and for devising integrated policy strategies for promoting compliance with the provisions.

Consideration should be given to the following steps and measures to strengthen the web of prevention.

- Universalisation of international legally binding instruments.
- Enhancing the interaction between the existing international mechanisms for multilateral negotiations with relevance to biosafety and biosecurity.
- Creating platforms for promoting action in support of the web of prevention for biological risk management.
- Developing integrated approaches for the national implementation of all elements of the web of prevention for biological risk management.

*Breaking the Silo Effect – Multi-sectoral Collaboration at Global Level to Operationalize One Health – The Ohio State Global One Health initiative model – presented by Tigist Endashaw Bealem, Ohio State Global One Health initiative, Ethiopia.*

The challenge to a One Health approach is how to build capable professions that can influence policy and sustain impact. Cognizant of this huge gap in capacity, in 2009 the Ohio State University established a regional Global One Health Initiative (GOHi) in Addis Ababa, Ethiopia. Its mission is to expand capacity for a One Health approach via applied education, training, research and outreach to more efficiently and effectively address causes and effects of diseases at the interface of humans, animals, plants and the environment. It has five strategic pillars: training capacity; research & implementation science capacity; outreach & extension capacity; resource stewardship; and financial stewardship.

Multi-sector collaboration is key to effectively address the challenges of the One Health approach. The GOHi model aims to showcase the benefits of such an approach in the control and prevention of zoonotic, emerging and re-emerging infectious diseases.

Breaking the silos involves internal and external aspects. It also necessitates a cohort of higher education leaders who are equipped to think multi-disciplinarily. Universities therefore need to develop programmes that draw from seemingly disparate academic disciplines and begin integrating a community engagement element and experiential learning component to academic learning.

The ongoing COVID-19 pandemic has exposed the fragile capacity and uncoordinated response of countries. It has shown that there is an immediate need for implementation of collaborative efforts to mitigate and minimize the long-term impacts by using integrated models such as the Global One Health paradigm.

However, major challenges lie ahead. These include resistance to change; high turnover of staff within institutions; misinterpretation and miscommunication; lack of transparency; and limited resources. Despite these challenges, GOHi has brought together ministries, research centres, regulatory bodies, universities, policymakers, national and international health institutes and community stakeholders from all three sectors to work on capacity building across a range of areas.

Breaking the silo system of traditional working conditions, GOHi attracted 13 Ethiopian government organisations to develop a roadmap for control of rabies in targeted regions of the country. It also efficiently executed the CDC GHSA agreement: training, coordinating and running different projects focused on zoonotic disease prevention and AMR.

GOHi coordinated and implemented the annual One Health Summer Institute in eastern Africa which has trained more than 1760 people since 2012 and carried out 21 One Health workshops. Another programme is the One Health Eastern Africa Research and Training (OHEART) capacity which started in 2010 to address vector-, food- and water-borne issues. It has led to 11 PhDs.

GOHi also conducted a rapid study evaluating non-pharmaceutical interventions in Ethiopia to evaluate the country’s preparedness for COVID-19. It is currently conducting active surveillance for suspects of COVID-19 to support the Ethiopian government’s endeavour to mitigate the pandemic.

*How could we halt the international bushmeat trade, a threat to biodiversity and public health? Policy perspectives and recommendations following a 2-year study on bushmeat trade from Africa to Belgium – presented byAnne-Lise Coralie Chaber, University of Adelaide, Australia.*

Bushmeat or wild meat is defined as the flesh of any wild animal that is hunted by local communities for subsistence and trade. It is estimated to be worth 10–20 billion USD per year. Bushmeat is an essential source of protein in rural areas in Africa, Asia and Latin America. However, the growing demand for bushmeat in urban centres is a threat to biodiversity and public health. The high financial rewards for catching, transporting and selling bushmeat, combined with the low risk of being caught and punished, is driving bushmeat trade into Western countries to make it a global business.

Two European regulations are in place: a wildlife trade regulation and a food safety/human health regulation. However, meat that enters Europe illegally often travels across borders without checks. There is no data on the intra-European trade of bushmeat and the trade network used.

To gain better understanding of bushmeat being illegally imported into Europe, a study was conducted at Brussels airport, 2017–2018. After luggage checks, the species involved were genetically identified by molecular tracing using the bushmeat DNA database, along with any alien pathogens or species carried. A wide spectrum of species was seized at the airport, many of which are identified by CITES as endangered.

A total of 284 seizures yielded 794 kg of bushmeat, of which 647 kg originated from Africa. The average weight of bushmeat per flight was recorded, in order to estimate the total rates of bushmeat imported into Belgium. The estimation of 46.5 tons per year corresponds with previous studies indicating 273 tons/year coming into France (2008), and 8.6 tons/year arriving in Switzerland (2008–2011).

This shows that the trade in illegal bushmeat in the EU is significant, but little data is consistently available for air cargo, cargo ships, mail or rail. There is also no information on the possibility of an organised criminal network in Europe, nor any intro-European trade information. And currently no prosecutions are being carried out under any of the existing regulations.

In terms of recommendations, active support from the air transport sector is needed. Studies have to be carried out on a European scale to estimate the sale and consumption of bushmeat in the EU, as well as the sociological components and the trade routes. It’s also necessary to assess the risks to public health and animal health linked to this trade.

The complexity and scale of the problem requires the setting up of global partnerships involving all private and public stakeholders. Two initiatives have been taken. One of them – the Buckingham Palace Declaration – has currently been signed by 120 stakeholders. Greater support from the air transport sector is essential. Moreover, an awareness raising campaign and enforcement efforts are needed as well as data and coordination at European level.

*UK: One Health in practice – presented by Christine Middlemiss, Department for Environment, Food and Rural Affairs, United Kingdom.*

In terms of disease risk mitigation, the UK government’s objective is to protect the nation’s biosecurity by responding to threats robustly, putting effective mitigation or eradication measures in place, and managing risks. The Biosecurity Continuum covers pre-border activities, border activities, and in-country activities.

After the 2007 ft & mouth disease outbreaks in the UK, the Anderson Review recommended instituting a system of regular, consistent, systematic risk assessments for new and re-emerging animal-related threats. A number of tools for risk identification have been developed, and the risk leads are fed into the ETHiR tool (Emerging Threat Highlight Report) which assesses and evaluates them against four key indicators (the potential impacts on public health, animal welfare, economic & society health, and trade).

Potential actions are then considered by a group of experts. Risks are fed into the system from all across the animal health spectrum in the UK. International disease changes are also monitored that may impact animal health and human health. This work is undertaken monthly.

Surveillance within the UK is a key part of the work. This includes epidemiology risk assessments, monitoring (e.g. rabies in bats and parasites in foxes), and statutory surveillance (e.g. *Salmonella* and *Brucellosis* in livestock), all of which are brought together in the UK Surveillance Forum. Risks of potential human impact are taken to the Human Animal Infections and Risk Surveillance (HAIRS) Group where experts discuss and understand them and advise officials and ministers on whether the risk situation is stable, deteriorating, improving, or undetermined. The detection of the Schmallenberg virus which is associated with disease in cattle, sheep and goats in Europe went through this process.

A One Health approach is taken to AMR, where the UK livestock industry has – without legislation – reduced antibiotic sales in animals by 53% over the past four years. The next focus is on reducing endemic disease levels in animal populations and working to understand the role of the environment in AMR. The UK’s collaborating centres and reference laboratories have been recognised by FAO, WHO and OiE in the context of AMR.

COVID-19 has clearly emphasised the need and importance of a One Health approach. Here the key priority is to preserve human health while maintaining the food chain. Lessons learned include the greater use of technology for communication and decision making; using effective processes already in place; improving the global One Health focus and coordinating horizon scanning; and ensuring key roles for professional disciplines with well-evidenced animal health and scientific advice to ministers. Also essential is building partnerships with international colleagues.

## Operational frameworks

*Walking the Talk: One Health Operationalization in the Free State, South Africa – presented byClaudia Cordel, ExecuVet Pty Ltd, South Africa.*

A five-year One Health research project was recently completed looking at the ecology and epidemiology of Rift Valley Fever on 363 farms in central South Africa. Climate, weather, vegetation, soil, and vector abundance and succession were monitored on 23 permanent field sites along with samples from live animals. Joint analysis of data from wildlife and livestock was carried out.

Looking at disease through an integrated approach is essential to the success of eradication or effective control to minimize disease effects on livelihoods, local and regional economies. The role of wildlife needs to be elucidated for Rift Valley Fever control strategies in South Africa. An ongoing One Health approach to disease monitoring and surveillance at the wildlife/livestock interface will be beneficial to public health disease prevention.

In raising community awareness and education around risk behaviours and mitigation of risk, and providing training opportunities through a variety of tools, booklets, and high-level workshops on the risks of arboviral zoonotic diseases and associated risk reduction activities, the One Health approach establishes a foothold in the new generation of leaders and communities alike.

It is still not understood which animals play a role in the maintenance of the Rift Valley Virus, or the role of the mosquito vector. To date no virus has been detected in adult or larval mosquitoes in the study area.

Among guidelines to consider are the need for synchronization of standard operating procedures for zoonotic diseases for surveillance and pathogen detection, improved networking between field and laboratory teams, and establishment of feedback mechanisms to communities to facilitate their engagement in disease reporting and control. Applied and in-depth trainings such as the PREDICT workshop help strengthen field-based skills and enhance awareness of pathways for integration of zoonotic disease prevention, detection and response measures in the surveillance system.

The One Health research approach provides a coordinated, resource efficient and multifaceted framework. The multi-disciplinary data collected through the One Health framework approach will better inform future integrated reporting and policy decision-making within optimal resource allocation. This improved data enables informed prioritization and identification of optimal potential intervention points in national and global objectives to improve disease control efforts.

*One Health Units for Humans, Environment, Animals and Livelihoods (HEAL) to ensure healthy people derive their livelihoods from healthy livestock in a sustainably managed environment – presented by Diana Onyango, Veterinaires Sans Frontieres - Suisse, Ethiopia (VSF-Suisse).*

In the Horn of Africa, over 30 million pastoralists face challenges such as inadequate access to human and animal health services, inadequate institutional capacity and infrastructure, vulnerability due to recurrent droughts, and increased competition for resource and associated conflicts.

The Humans, Environment, Animals and Livelihoods (HEAL) project aims to support resilience of drought and conflict-affected pastoralist communities in the area, by ensuring healthy people derive their livelihoods from healthy livestock in a sustainably managed environment. The strategy aims to reshape health service delivery in the form of One Health Units (OHUs) through a participatory, context-specific, coordinated and integrated approach, and to strengthen the essential health services at local level. The project is currently in its inception phase and involves alignment with relevant national strategies in Ethiopia, Somalia and Kenya.

The project started by mapping the One Health policy context and undertaking a needs assessment at the national level in areas such as governance and management; networks and partnerships; One Health capacity development; surveillance, preparedness and response; communication and advocacy; operational research; and monitoring and evaluation.

The communities were fully engaged through a multi-stakeholder innovation platform to understand the needs of communities directly and identify the best modality to implement OHUs and define the services they offer. Studies and surveys were carried out to obtain context analysis, including mapping and/or validation of the livestock migration routes, animal and health service delivery, and anthropology research. Findings included:

- Poor state of the health services.
- Remoteness of the facilities leading to logistical challenges.
- Poor infrastructure and physical barriers hampering accessibility of health services.
- Inadequate funding and resources of government-run facilities.

This enabled a variety of needs to be addressed such as rehabilitation of the health facilities; training of the frontline workers; improved inter-sectoral integration and coordination; improving access to supplies and drugs; strengthening public-private partnerships; and establishing village/district One Health platforms.

Mapping of the livestock migratory routes enabled the identification of next steps in participatory rangeland management, such as community governance of rangelands; rangeland management; relations with neighbouring communities; and relations with government and traditional systems. Key gaps and next steps were identified such as supporting rangeland health and rehabilitation, improving existing management plans, integrating livestock health into rangeland management plans, and strengthening rangeland management institutions.

Anthropology research was also carried out and indicated the impact of the religious leaders in sickness, the importance of treating common livestock diseases at all costs, the belief that animal diseases are “imported” by migrants, and that communities do not perceive climate change as a crisis but a temporary variation in weather patterns.

Eight OHUs are to be established in Phase 1, starting November 2020, to be managed and run by the existing public service providers.

In addition, a Regional Community of Practice is to be established to build a community of One Health practitioners and organisations. This will be through providing platforms where information can be shared, lessons can be learned, and people can exchange ideas on how to best use the One Health approach. This will be via a website, webinars, communication projects, and a social media presence.

*A One Health multi-sectoral national strategy for Brucellosis prevention and control in Kenya - presented by Mark Nanyingi, Institute of Infection and Global Health, Department of Epidemiology and Population Health, University of Liverpool, United Kingdom; School of Public Health, University of Nairobi, Kenya.*

An integrated One Health intervention strategy has been developed to prevent, control and if possible eliminate (by 95%) *Brucellosis* in Kenya. It’s based on a multi-sectoral collaborative approach and principles; livestock vaccination; surveillance, early detection and notification of the disease; proper intervention measures; and community engagement.

Key strategic objectives were developed. These are to harmonize appropriate legal/policy frameworks; to institutionalize *Brucellosis* testing among humans; to strengthen laboratory capacity; to enhance advocacy, communication, and social mobilization; and to mobilize resources for implementation of the *Brucellosis* control strategy. A full stakeholder developmental process was developed, leading to a comprehensive draft document which has undergone an extensive expert review process.

A *Brucellosis* situational (SWOT) awareness was carried out, along with integrated animal-human surveillance, and a study into the socioeconomic impacts and public health burden of *Brucellosis*. Challenges exist in developing diagnostic approaches which are being addressed. This will help direct and focus on the appropriate treatment guidelines for human *Brucellosis*.

The pre-implementation phase (2019–2020) included the establishment of national and subnational taskforces, along with guidelines for operationalization of the strategy. The implementation phase (2019–2038) incorporates a four-step approach, with each step aiming to see a declining level of human and animal *Brucellosis* and an enhanced capacity of national labs. Implementation will be carried out in regions of Kenya, starting with those where *Brucellosis* is most prevalent.

In terms of achievements and next plans, applied and operational research for implementation is ongoing in the areas of risk factor analysis, spatiotemporal modelling, vaccine uptake, and diagnostic algorithms. The National Brucellosis Strategy was intended to be launched in October 2020 but was delayed due to COVID-19. However, post-COVID the strategy will be launched and moved forward very quickly, with development of guidelines on joint outbreak investigation, risk communication, human case management, and vaccination coverage planned for 2021.

*One Health Stakeholder Initiatives – presented by Jomana Musmar, US Department of Health and Human Services, United States of America.*

In 2019 the WHO identified the top ten threats to global health, many of which are addressed by the US Department of Health and Human Services (HHS) across the One Health spectrum. Its mission is to provide strategic leadership and management while encouraging collaboration, coordination and innovation among federal agencies and stakeholders, to reduce the burden of infectious diseases. The department oversees key public health offices and programmes, a number of presidential and secretarial advisory committees, and ten regional health offices across the US.

The Office of Infectious Disease and HIV/AIDS Policy (OIDP) focuses on HIV/AIDS and STIs; blood and tissue safety and availability; vaccines; viral hepatitis; tick-borne diseases; emerging infectious diseases; antimicrobial resistance; and management of Federal Advisory Committees. OIPD coordinates four critical national strategies: HIV Plan, Hepatitis Plan, STI Plan, and Vaccine Plan, all of which have been or are currently being updated.

PACHA is the Presidential Advisory Council on HIV/AIDS, It provides advice, information and recommendations to the HHS regarding HIV prevention, treatment, and care. A key deliverable is the “Ending the HIV Epidemic” initiative. In addition, the “PACHA to the People” series convenes in high-priority areas, engages the general public, and visits local community organisations.

The Advisory Committee on Blood and Tissue Safety and Availability advises the HHS Secretary on a range of relevant policy issues. Most recently it has studied the impact of COVID-19 on blood supply and on future pandemics.

The Tick-Borne Disease Working Group provides a report on recommendations for the federal response to tick-borne disease prevention, treatment and research, as well as how to address gaps in these areas.

In terms of AMR and One Health, two bodies have been created: the Presidential Advisory Council on Combating Antibiotic-Resistant Bacteria (PACCARB) and the Interagency Task Force on Combating Antibiotic-Resistant Bacteria (TF-CARB). A One Health approach is emphasized and utilized for all activities.

The Division of Vaccines is responsible for coordinating and ensuring collaboration among the many federal agencies involved in vaccine and immunization activities, and also helps to promote vaccination. The National Vaccine Advisory Committee (NVAC) recommends ways to achieve optimal prevention of human infectious diseases through vaccine development, and provides direction to prevent adverse reactions to vaccines. It is involved in the “Catch Up To Get Ahead” campaign to promote child immunization in the face of the current significant decline.

## Risk reduction frameworks/Global Health security

*A regional approach to health emergencies: The ASEAN EOC Network – presented byChee Kheong Chong, Ministry of Health Malaysia, Malaysia.*

The Association of Southeast Asian Nations (ASEAN) was formed in 1967 and currently includes ten Member States. It is based on the three pillars of Political-Security, Economic, and Socio-Cultural communities; the latter including the health sector. The need for ASEAN to respond to natural disasters as a region was acknowledged in 2011 with the establishment of the ASEAN Coordinating Centre for Humanitarian Assistance (AHA). Humanitarian assistance and disaster relief is further provided by the ASEAN Centre of Military Medicine (ACMM).

However, regional response for health events other than natural disasters was not established. The health sector recognizes the importance of a dedicated structure to address regional health needs, irrespective of the type of hazards or emergencies (natural or intentional). This led to the establishment of the ASEAN EOC (Enhancing Operations Centre) Network.

Phase 1 was to build trust among ASEAN Member States and initiate the sharing of information with some degree of training. Phase 2 is to build on phase 1 as well as to establish a structure or procedure to respond.

The main areas currently underway are improving laboratory capacity, identifying and preparedness for mounting a medical & public health response and coordination mechanism. Other areas will include risk assessment and risk communication, and the network is trying to develop an automated Internet based surveillance system. A number of key accomplishments have already been achieved:, including COVID-19 early response through information sharing among the network and partners.

The ASEAN EOC Network is a work in progress to develop an entity capable of responding to an all-hazards threat to medical and public health. Ultimately, it should be capable of automatic internet surveillance, coordination among the 10 ASEAN Member States, and an ability to organize a joint response.

*ASEAN Architecture in Responding to All Hazards and Emerging Threats: ASEAN Health Sector Perspective in Multi-sectoral and Multi-stakeholder Cooperation – presented byFerdinal Moreno Fernando, ASEAN Secretariat, Indonesia.*

ASEAN has experienced high-impact infectious diseases that threatened the public health and functioning of its communities. These are the Severe Acute Respiratory Syndrome (SARS) in 2001; the Highly Pathogenic Avian Influenza (HPAI) in 2003; the pandemic caused by H1N1 Influenza Virus; the threats of Ebola Virus in 2014; the Middle East Respiratory Syndrome (MERS COV) in 2015; and the Zika Virus in 2016.

To combat such threats, ASEAN has conducted great efforts to enhance its whole-of-government and whole-of-system approaches in the detection, mitigation/prevention and response to combat infectious diseases. Efficient and effective coordination mechanisms, and addressing relevant capacity gaps have been prioritized to address critical areas for investing appropriate efforts and support.

The ASEAN Post-2015 Health Development Agenda (APHDA), through the cluster approach in strategically responding to all hazards and emerging threats, is enhancing multi-sectoral partnerships; engendering health in ASEAN policies and cooperation; narrowing the development gaps among AMS, and establishing appropriate mechanisms of coordination and collaboration.

Building on the gains from the regional work programme on communicable and emerging infectious diseases and relevant lessons learned in the past years, and regional policies, the ASEAN Health Sector enhanced its capacities in preventing, detecting and responding to all hazards and emerging health threats. These include, among others:

- Revising the regional health governance and implementation mechanism.
- Prioritizing the health-security interface.
- Focusing on value addition of regionwide emergency operation centre (EOC) networking for public health emergencies (PHE).
- Enhancing biosafety/biosecurity, and laboratory capacities.
- Enhancing disease surveillance cooperation.
- Addressing anti-microbial resistance.
- Enhancing regional capacity in big data analytics for disease surveillance.
- Further synergizing with disaster health management initiatives.

The Mitigation of Biological Threats (MBT) programme started in 2013 and started playing a key role in the response to COVID-19 as early as 3 January 2020.

*Joint External Evaluation of Finland: Enhancing health security through a comprehensive whole-of-government approach - presented by Simo Nikkari, Professor and Director at Centres for Biothreat Preparedness and Military Medicine, Finland.*

A JEE was conducted in Finland in March 2017 by experts from the WHO and other international organisations. The JEE is an iterative process to identify and fill gaps in health security. It evaluated 19 technical areas in four main topic areas: prevent, detect, respond, and other IHR related hazards and points of entry.

The Finnish strategy to secure vital functions for society is described in Finland’s Security Strategy for Society (2017). The strategy is monitored by the Security Committee which oversees the coordination of national security including the health risks posed by the malicious use of biological agents. A multi-sectoral steering group is in place for implementation of the national action plan.

The report of the Finnish JEE is available online and the findings were published in the April 2018 issue of the WHO Public Health Panorama Journal. In the findings, Finland’s strong public health capacity was acknowledged, along with the potential to share its knowledge and skills to support other countries in capacity building to promote global health security. The report recommended three high-level actions:

- Plans, policies, strategies, regulations and legislation should continue to support the implementation of IHR, One Health policy, and a comprehensive health security approach with adequate provision of resources in each technical area.
- In the absence of major real events, there is a risk of complacency, thus it is necessary to continue advocacy in investing in IHR.
- High levels of collaboration with multi-sectoral partners should be complemented with a clear chain of command and decision-making in structures.

The JEE recommendations have been carefully studied for integration into the existing national action plan for health security and are gradually being incorporated into other major policies, strategies, action plans and legislation.

*Pandemic Preparedness Planning in Peacetime: what is missing? Presented byAb Osterhaus, Research Center for Emerging Infections and Zoonoses, University of Veterinary Medicine, Hannover, Germany and chair One Health Platform, Germany.*

Pandemic preparedness includes a number of critical elements such as early warning systems; pathogen discovery and characterization platforms; diagnostic platforms; mathematical models; animal models in BSL3 facilities; clinical trial platforms; non-pharmaceutical intervention and treatment strategies; and pharmaceutical intervention strategies such as antiviral platforms, vaccine platforms, and Biological Response Modifiers (BRM) platforms. Significant investments are necessary in many of these areas to ensure optimal health security in the future.

In regard to COVID-19, countries such as China, South Korea and Singapore responded extremely quickly to eliminate it, whereas the EU seemed to have 28 different policies which focused more on living with the virus than eliminating it. This was despite early warnings of pre-symptomatic infectiousness, which were unfortunately not taken seriously.

Early warning systems include syndrome- and lab-based surveillance; reporting systems; lab-based diagnosticians; experts in rapid molecular virus characterization; and national and international reporting systems. Progress is being made in these areas, and WHO continues to be instrumental in promoting early warning systems. China did an excellent job to characterize the virus and develop diagnostics rapidly. A key concern is hospital and ICU capacity, but that shouldn’t be the focal point, it should be on getting rid of the virus.

In regard to COVID-19 vaccines, 35 are currently in phase 1 clinical trials, 14 in phase 2, and 11 in phase 3, so the industry is moving extremely quickly, although it’s essential that any vaccine is totally safe. The question is how much vaccine is likely to be available by end of the year? Predictions are difficult to make, and it’s likely that vaccines will not be available in large quantities before summer 2021.

## Civil society participation/risk communication

*Community mapping and engagement to co-create a One Health conceptual diagram of under-5 (U5) infections in urban slums for the Childhood Infection and Pollution (CHIP) Consortium - presented by Prof Monica Lakhanpaul, UCL Great Ormond Street Institute of Child Health, United Kingdom.*

The Childhood Infection and Pollution (CHIP) Consortium is a multi-country and multi-disciplinary endeavour that aims to reduce the burden of infection and antimicrobial resistance (AMR) in children under five. The focus is on children living in resource-deprived urban communities. The under-fives are among the world’s most vulnerable people, because growth and development in early childhood shows exceptional environmental sensitivity, presenting a critical window to intervene to counter the effects of pollutants. The work uses co-produced behaviour change and community upgrading interventions. Work of the consortium is ongoing in Chile, Peru, India, Nepal and Indonesia.

The CHIP Consortium considers that the One Health concept is vital to get a more comprehensive understanding of the problem and potential solutions than would be possible with siloed approaches. Community-focused research is carried out, along with collaborations with local, state and national public and private sector actors.

Between September and December 2019, a mixed methods approach was used, with film, geo-tagged pictures and qualitative interviews to conduct a feasibility study. This was to observe potential infection linkages as well as social-cultural aspects of each community. Community champions and local community engagement teams were recruited and trained to support the field work. They used a variety of methods in slum communities in Chile, Indonesia and India. Transect walks involved walking around communities and noting potential risks. Social mapping involved people from the communities working out where the resources, shops, and primary health facilities are in their community. Social mapping expands on the transect walk to understand the social geography and community structure of slum sites. All geographical data are then uploaded onto OpenStreet maps.

Interviews with community leaders, community champions and parents of children under-five were conducted to ask if sample-taking was acceptable (from the children, animals and the surrounding environment). With safeguards (e.g. a local doctor accompanying the researchers) a high acceptability of invasive procedures was observed across the three countries. The parents were also asked about infections suffered by the children.

All of the information was then collated into a conceptual map showing human, animal and environmental factors to indicate the linkages between many different factors in the child’s environment.

*Establishing knowledge, attitudes and practices of Australian general practitioners and veterinarians to develop interventions to improve preparedness for zoonoses related health emergencies – presented by Sandra Steele, University of Sydney, Australia.*

In the event of a zoonotic health emergency, the effectiveness of response depends on the knowledge and experience of those involved, such as general medical practitioners and veterinarians. They are strategically positioned to identify index cases of emerging diseases, and to play a continuing role in surveillance and management of ongoing diseases in health emergencies. Cross-professional collaboration and cooperation is therefore essential, as is an application of One Health.

To determine the preparedness of GPs and veterinarians in Australia, as well as ascertain their attitudes and practices in regard to One Health, an Australia-wide cross-sectoral survey was conducted to determine experience, concern, confidence, and practices with regard to zoonoses. Responses were received from 528 GPs and 605 veterinarians.

Certain gaps were identified. GPs had less experience and lower levels of concern and confidence with zoonoses than veterinarians. Rural practitioners from both professions showed greater concern and confidence with zoonoses. There was a lack of effective One Health literacy and practices in both groups.

Some of the reasons for these gaps are clear. Veterinarians have greater training in zoonoses, epidemiology and biosecurity in their degree, which probably explains their heightened awareness. They spend much of their working days focusing on animal health and they have certain legal obligations too. The lack of One Health knowledge and practices is likely to be due to a combination of factors: a varying level of knowledge about zoonoses, poor understanding of the skills of other professionals, no established relationships between professional groups, and an absence of established processes to implement One Health in clinical practice.

Addressing the gaps and improving practices to manage zoonoses is the next step forward.

Encouraging interaction between GPs and veterinarians and improving understanding of each other’s professional skills and roles can build capacity and capability for practitioners. Running regional events can create opportunities to develop these relationships and hopefully facilitate One Health practices in their areas.

COVID-19 has shown that effective emergency disease management cannot be developed on-the-fly when processes and structures are inadequate. The pandemic has highlighted the need for all primary health professionals to be well prepared in the face of a health emergency. It’s therefore imperative to work towards the establishment of a structured cross-professional interface by establishing and consolidating pathways to facilitate effective collaboration. Strategic changes and interventions are needed to ensure that frontline veterinary and medical practitioners understand the benefits of and are accustomed to working within an established One Health framework. This will ensure, in the event of a zoonotic-based health emergency, that better tools will be in place to manage and mitigate disease impact and spread.

However, other obstacles exist. Governments worldwide have failed to prioritise funding of One Health and emerging disease research. Health professionals are stuck in their own silos. One Health is often a neglected area within medico-legal frameworks. And veterinarians are faced with constraints when investigating diseases, such as inadequate funding.

*Risk practices for bovine tuberculosis transmission to cattle and livestock farming communities living at wildlife-livestock-human interface in northern KwaZulu Natal, South Africa presented by Petronillah Rudo Sichewo, University of Pretoria, South Africa; Midlands State University, Zimbabwe.*

Increasing agricultural activities has led to the encroachment of human activities in conservation areas in South Africa, resulting in wildlife, livestock and humans sharing the same ecosystems. Transmission of bovine TB between wildlife and cattle is bidirectional, involving both spillback and spillover, and has been diagnosed in 21 wildlife species in South Africa. Bovine TB mainly causes extra-pulmonary TB in humans, with transmission via consumption of contaminated animal products, and direct contact (aerosol from infected animals).

A study was conducted in northern KwaZulu Natal among communal cattle farmers with bovine TB tested herds to assess local knowledge of bovine TB and investigate associated risk practices in cattle and in people coexisting with wildlife. Four focus categories were identified: dip tank committee members; heads of households; women from cattle farming households; and male and female cattle herders. Each group had 10–14 participants. Data analysis focused on six themes:

- Knowledge of bovine TB.
- Food preparation and consumption practices.
- Cattle slaughtering and meat inspection.
- Criteria for accepting animals into herds and veterinary services.
- Introduction of cattle into herds by communal farmers.
- Cattle-cattle/cattle-wildlife interactions.

A high degree of consumption of undercooked meat and raw milk was found, and a lack of protective measures during slaughtering of cattle. Risk practices for bovine TB transmission to cattle were identified as the sharing of pastures with other herds and wildlife, the introduction of animals into herds without bovine TB pre-movement testing, and the sharing of watering points with other herds and wildlife.

Participants in the survey showed a high awareness of bovine TB in cattle and its transmission to humans, but paradoxically this was coupled with poor preventive practices, which in turn are linked to the socio-economic status of farmers. Food preparation and consumption is influenced by habits, beliefs and socio-cultural factors. Male cattle herders are at greater risk due to unprotective practices during the slaughter of animals and the consumption of raw animal products. The free movement of animals in communal farming was seen to be a common practice, while porous boundaries led to contact between wildlife and cattle, resulting in the exchange of diseases.

*Community Engagement and Longevity of Zoonotic Disease Knowledge in Rural Uganda – presented byA Mixed-Methods Approach: Sarah Paige, CORE Group, United States of America.*

The Kibale National Park in Uganda has experienced increasing human population settlement, and at the same time increasing conversation success. For 15 years the Kibale EcoHealth Project has been conducting research in the communities in the park to understand the human-animal interface and the interactions that could facilitate spillover in zoonotic disease. Methods included focus group discussions, interviews, guided walks, and two knowledge, attitude and practice surveys.

The results of qualitative data collection indicate a high level of knowledge about zoonotic disease in the communities along the Kibale National Park, which makes sense given the longevity of the research and the intensity of conservation in the area. Respondents reported that human livelihood activities expose individuals to wild animals. This was interaction driven by wildlife, not vice versa. The biggest issue relating to conservation goals is crop-raiding and the inability to gather firewood. Again, wildlife was driving human-animal interactions through incursions into compounds and gardens near the park.

Participants suggested that researchers should strive to address their priorities. They appreciated the presence of researchers but had grievances that the results were not shared back. Research should therefore strive to address community priorities such as education, agricultural development, and access to reliable healthcare.

It’s clear that research engagement impacts communities. A modest, inexpensive intervention included as part of a research programme can have lasting effects on knowledge. Participants were able to report a high number of protective behaviours: however, they are unable to implement those behaviours due to structural and environmental limitations.

The research led to four conclusions:

- It’s important to realise the impact that research programmes have on communities more broadly.
- By incorporating modest interventions, zoonotic disease programmes could have sustained impact on participant knowledge.
- Participants’ capacity for implementation of preventive behaviours is limited by circumstances.
- Community-centric research programmes that address participants’ priorities are fundamental to discovering emerging zoonoses.

*Intensive Livestock Farming: Is the risk for human health really the problem? Perceptions of scientists, residents and farmers – presented by Valerie Eijrond, Amsterdam UMC, Vrije Universiteit Amsterdam, Department of Public and Occupational Health, The Netherlands.*

The Netherlands has the highest livestock density of EU Member States and is Europe’s largest agricultural produce and food exporter. On a surface area of 41,500 km^2^, 17 million people live together with 100 million broilers and laying hens, 12 million pigs, 4 million cows, 800,000 sheep, and 500,000 goats. This has led to much societal debate about the future of intensive livestock farming, in particular around concerns about human health and other issues. For example, research pointing out potential health risks such as pneumonia and goat farms has led to Dutch provinces stopping issuing permits for goat farms. Another relevant news item concerns COVID-19 outbreaks in mink farms.

Various stakeholders such as scientists, farmers and citizens are involved in discussions, but they often have opposing perspectives on livestock farming. Consequently, public meetings rarely result in fruitful discussions or accepted compromises. A collaborative approach among a variety of stakeholders is necessary for policy development. However, for them to be fruitful, communication, trust and mutual commitment are crucial. Therefore it’s essential to identify the core issues at stake.

A mental models approach involves exploring the similarities and disparities of scientific experts and non-experts regarding knowledge, beliefs and concerns on human health and intensive livestock farming. This was expanded to residents and farmers and their perceptions of intensive livestock farming and human health, and identified their major concerns.

It’s clear that residents hold negative views on livestock farming and see only one solution: livestock shrinkage and downsizing the industry. In contrast, farmers understandably have positive views of intensive livestock farming. The central problem is that these contrasting views are both valid at the same time.

Three types of evidence bases are vital for policymaking: a balanced interplay of scientific facts, practical experience, and political judgement. Scientific experts are often inclined to inform the public about the health risk assessment of intensive livestock farming, but a technocratic approach does not necessarily address the concerns of residents, who are often more concerned about other health issues, such as odour, noise and traffic safety, and the impact on nature and animal welfare. These issues are insufficiently addressed in communication, negatively influencing the reception of risk communication messages.

Secondly, the diverging concerns for human health is only one aspect of the problem. Intensive livestock farming is a cross-cutting issue touching multifaceted concerns besides human health risks. Different disciplines need to work together. A policy that may address one concern such as health may lead to other concerns in another discipline such as animal welfare. To address the problems associated with intensive livestock farming requires communication and collaboration across multiple stakeholders.

